# Aneurism

**Published:** 1828-02

**Authors:** 


### ANEURISM.

### *Axillary Aneurism of the Right Side. Operation of Tying the Subclavian Artery.*

The following appears to be one of the most dexterously performed and successful operations of the kind on record.

William Weston, æt. thirty-eight, a stout and very muscular man, was admitted November 28, at Guy's Hospital, under the care of Mr. Bransby Cooper. About three months ago, he felt a slight pain and numbness just below the middle of the right clavicle, which extended down the arm to the fingers: this occurred once or twice a week, increasing in severity each time. At the end of a month, the paroxysms had become so violent that, during their continuance, he was obliged to desist from his ordinary occupation—that of a wood hawker. In nine weeks from the first attack, his attention was directed to a slight fulness upon the upper and fore part of the chest. Sixteen years ago he worked very hard, excavating a dock; he drank freely at that time, but has lived very temperately for the last few years.

Upon examination, a large pulsating tumor was perceived in the axilla of the right side, extending to the fore part of the chest, and producing a fulness, about the size of an egg, between the subclavius muscle and clavicular portion of the pectoralis major: the tumor could be traced so far back as the edge of the latissimus dorsi; the base could not be distinctly felt; handling the swelling increased the pain; a very slight pressure on the subclavian artery completely stopped the pulsation in the tumor. The arteries of the arm were uninfluenced by the aneurism, the pulsations at both wrists corresponding, which, however, were unusually feeble. The numbness and pain had become so great as entirely to deprive him of rest. He was ordered a little aperient medicine a day or two prior to the performance of the operation, which took place on Tuesday, December 4th.

The patient was placed upon a long table, in the horizontal posture,* with the shoulders slightly elevated, the head directed to the left side, the right arm forcibly drawn down, and the shoulder depressed. The integuments covering the clavicle were stretched upon the chest, and the operation commenced by making an incision about three inches long, which laid bare the edges of the sterno-cleido-mastoideus and trapezius muscles; when the skin, from its natural elasticity, retracted, so as to leave a free opening above the clavicle, exposing the superficial cervical fascia, the platysma myoides being perfectly divided. The centre of this incision was met by a vertical cut, in the direction of the posterior edge of the sterno-cleido-mastoideus. The external jugular vein was dis-

* Mr. Cooper here remarked upon the necessity of the horizontal posture, for the entire deprivation of all muscular action, every fixed point being thus taken away.

tinctly seen on the inner side. The anterior edge of the scalenus anticus muscle was next laid bare by clearing away (with the sharpened handle of the knife) the condensed cellular texture, during which process the omo-hyoideus was exposed, and the artery seen immediately emerging from between the scaleni: it was easily cleared from the surrounding parts, so as to admit of the passage of the aneurismal needle. This instrument deviated from the one generally used, by forming a larger portion of the segment of a smaller circle, so as to occupy much less room. A single silk ligature was passed under the artery, and tied in the ordinary way: the vessel could then be seen pulsating between the heart and the ligature, although previously the pulsations could scarcely either be seen or felt. One end of the ligature being cut off close to the artery, the other was left at the external wound. The integuments were brought together by a single suture and adhesive plaster, and the right arm and hand were enveloped in flannel. The patient was then replaced in bed, having scarcely lost an ounce of blood: indeed, only one small artery was divided, and which ceased bleeding without being tied. The pulsation in the tumor entirely ceased upon the application of the ligature, and the sac soon became completely empty. The patient expressed great relief, and did well.

